# Therapeutic Potential of Anti-Interferon α Vaccination on SjS-Related Features in the MRL/lpr Autoimmune Mouse Model

**DOI:** 10.3389/fimmu.2021.666134

**Published:** 2021-11-17

**Authors:** Martin Killian, Fabien Colaone, Philippe Haumont, Carole Nicco, Olivier Cerles, Sandrine Chouzenoux, Pascal Cathébras, Nicolas Rochereau, Blandine Chanut, Mireille Thomas, Norbert Laroche, Fabien Forest, Géraldine Grouard-Vogel, Frédéric Batteux, Stéphane Paul

**Affiliations:** ^1^ Centre International de Recherche en Infectiologie (CIRI), Team Groupe Immunité des Muqueuses et Agents Pathogènes (GIMAP), Université de Lyon, Université Jean Monnet, Université Claude Bernard Lyon 1, Inserm, U1111, Centre National de la Recherche Scientifique (CNRS), UMR530, Saint-Etienne, France; ^2^ Internal Medicine Department, Saint-Etienne University Hospital, Saint-Etienne, France; ^3^ NEOVACS S.A., Paris, France; ^4^ Team Stress Oxydant, Prolifération Cellulaire et Inflammation, Institut National de la Santé Et de la Recherche Médicale (INSERM) U1016 Institut Cochin, Paris, France; ^5^ Institut National de la Santé Et de la Recherche Médicale (INSERM) U1059-Sainbiose, Université de Lyon, Saint Priest en Jarez, France; ^6^ Department of Pathology, Saint-Etienne University Hospital, Saint-Etienne, France

**Keywords:** Sjögren’s syndrome, vaccination, interferon a (IFN-a), adjuvant, kinoid

## Abstract

Sjögren’s syndrome (SjS) is a frequent systemic autoimmune disease responsible for a major decrease in patients’ quality of life, potentially leading to life-threatening conditions while facing an unmet therapeutic need. Hence, we assessed the immunogenicity, efficacy, and tolerance of IFN-Kinoid (IFN-K), an anti-IFNα vaccination strategy, in a well-known mouse model of systemic autoimmunity with SjS-like features: MRL/MpJ-Faslpr/lpr (MRL/lpr) mice. Two cohorts (with ISA51 or SWE01 as adjuvants) of 26 female MRL/lpr were divided in parallel groups, “controls” (not treated, PBS and Keyhole Limpet Hemocyanin [KLH] groups) or “IFN-K” and followed up for 122 days. Eight-week-old mice received intra-muscular injections (days 0, 7, 28, 56 and 84) of PBS, KLH or IFN-K, emulsified in the appropriate adjuvant, and blood samples were serially collected. At sacrifice, surviving mice were euthanized and their organs were harvested for histopathological analysis (focus score in salivary/lacrimal glands) and IFN signature evaluation. SjS-like features were monitored. IFN-K induced a disease-modifying polyclonal anti-IFNα antibody response in all treated mice with high IFNα neutralization capacities, type 1 IFN signature’s reduction and disease features’ (ocular and oral sicca syndrome, neuropathy, focus score, glandular production of BAFF) improvement, as reflected by the decrease in Murine Sjögren’s Syndrome Disease Activity Index (MuSSDAI) modelled on EULAR Sjögren’s Syndrome Disease Activity Index (ESSDAI). No adverse effects were observed. We herein report on the strong efficacy of an innovative anti-IFNα vaccination strategy in a mouse model of SjS, paving the way for further clinical development (a phase IIb trial has just been completed in systemic lupus erythematosus with promising results).

## Introduction

Sjögren’s syndrome (SjS) is a frequent and predominantly female (10:1) systemic autoimmune disease, affecting 0.01-0.3% of the adult population worldwide (1). Sicca syndrome (i.e. dry eyes and/or mouth), lymphocytic infiltration of the salivary and lacrimal glands and anti-Ro/Sjögren’s Syndrome A (SSA) and anti-La/Sjögren’s Syndrome B (SSB) autoantibodies are typical in SjS (2). Apart from these classical features, the clinical spectrum is broad with systemic (i.e. extra-glandular) manifestations in one third of cases (3, 4). SjS’s burden in terms of quality of life is well-known but the disease is also responsible for increased mortality with recent estimates of the adjusted standardized mortality ratio of 4.66 (95% CI 3.85 to 5.60) (5). Despite these worrisome elements, therapeutic resources are scarce, artificial tears and secretagogues only offer temporary relief, and no immunomodulatory treatment was superior to placebo in randomized controlled trials to date (6), partly because of the difficulty in the choice of the right outcome (7). However, a growing body of evidence indicates a major role for type 1 interferons (IFNs) in Sjögren’s pathogenesis (8), susceptibility (9), and disease activity/complications (10, 11), hence becoming a potential therapeutic target. Among these findings, the identification of a type 1 IFN signature – the overexpression of type 1 IFN-inducible genes – in the blood and salivary glands of more than 50% of SjS patients (9, 10), its correlation with disease activity and B cell activating factor (BAFF) – which is type 1 IFN-inducible – overexpression (10, 12), the description of potentially protective endogenous anti-IFNα autoantibodies in SjS (13), and the promising results of recent studies evaluating anti-B cells therapies such as the anti-BAFF mAbs, belimumab (14), alone or combined with rituximab (15, 16), or ianalumab (17) (whose phase III trial is about to start in 2021), as well as a preclinical trial with filgotinib (18), a JAK1 inhibitor (with effects on IFN pathways, including BAFF production), are noteworthy. We previously showed that an active immunotherapy strategy, IFN-Kinoid (IFN-K), made up of conjugated IFNα and Keyhole Limpet Hemocyanin (KLH), was successful in preventing systemic lupus erythematosus (SLE)-manifestations and improving survival in New Zealand Black and White (NZB/W) mice (19), by inducing neutralizing anti-IFNα antibodies (Abs). Phase I/IIa (20, 21) and IIb (22) trials, in human SLE showed encouraging results, allowing the pursuit of IFN−K’s clinical development.

Given the pivotal role of type 1 IFN in SjS and the urgent need for treatments (23), we investigated in a preclinical study using the MRL/MpJ-Fas^lpr/lpr^ (MRL/lpr) model, a well-recognized mouse model of systemic autoimmunity with SjS-like features (24), the potential benefits of the murine surrogate of the human IFN-K vaccine.

## Materials and Methods

### Mouse Model

The MRL/lpr mouse model is a well-recognized congenital model of SjS (25–27), whose autoimmunity was found to be particularly caused by an acquired mutation in the lymphoproliferation (lpr) gene, leading to a defect in Fas-mediated apoptosis of auto-reactive T cells, prompting the proliferation of double negative (CD4- CD8-) T cells [which were previously found to be involved in the pathogenic mechanisms leading to SjS (28, 29)].

### Preparation of the Immunogen

IFN-K was prepared by complexing murine IFNα3 (PBL Assay Science, Piscataway, NJ) with KLH, by aldehyde treatment, as previously reported (19, 30).

### Study Approval

All experiments received approval of the INSERM and Université Paris Descartes CEEA34 Ethics committee on Animal Experimentation, under the number 15-012.

### Experimental Protocol

**Figure 1:**
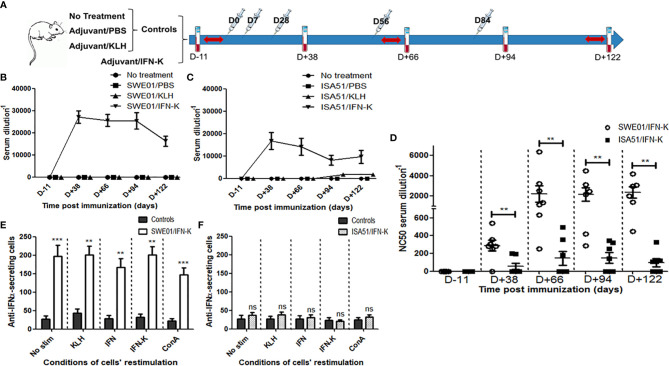
Immunological effects of IFN-K. **(A)** Within each cohort (n=26; SWE01 or ISA51), 8-week-old female MRL/lpr mice were not treated (n=6) or injected (syringes) with PBS (n=6) or KLH (n=7), referred to as Controls, or treated with IFN-K (n=7) depending on their group. Blood samples (blood tubes) were collected and Sjögren’s syndrome-like disease (red arrows) was monitored, once prior and serially after injections for a 122-day-follow-up. **(B, C)** Serum anti-IFNα Abs (ELISA) and their **(D)** anti-IFNα neutralizing capacity were monitored at different time points pre (D-11) and post-immunization (D+38, D+66, D+94, D+122) in all groups: no treatment (n=6+6), SWE01/PBS (n=6), ISA51/PBS (n=6), SWE01/KLH (n=7), ISA51/KLH (n=7), SWE01/IFN-K (n=7) and ISA51/IFN-K (n=7). Neutralizing activity was expressed as the serum dilution resulting in 50% neutralization of virus-mediated cell mortality (NC50). **(E, F)** Mononuclear splenocytes were isolated in all living mice (n=14 in SWE01-adjuvanted control mice, n=6 in SWE01/IFN-K mice, n=18 in ISA51-adjuvanted control mice and n=7 in ISA51/IFN-K mice) at sacrifice (D+122), and used for the quantification of anti-IFNα-IgG-producing cells. Proliferation of the harvested cells was stimulated under different conditions (no stimulation, ConA, KLH, IFNα or IFN-K, at a final concentration of 10 µg/ml), without noticeable impact. Error bars indicate mean ± S.E.M. Ns, **p < 0.01 and ***p < 0.001.

Two cohorts of 26 female MRL/MpJ-Fas^lpr/lpr^ (MRL/lpr, bred in our colony) mice were followed simultaneously (**Figure 1A**). One cohort, injected with ISA51 (Seppic, Paris, France), a water-in-oil adjuvant, was randomly divided into 4 parallel groups: NT (n=6), PBS/ISA51 (n=6), KLH/ISA51 (n=7) and IFN-K/ISA51 (n=7). The other cohort, whose adjuvant was SWE01 (University of Lausanne, Switzerland), an MF59-like oil-in-water emulsion, was also randomly divided into 4 parallel groups: NT (n=6), PBS/SWE01 (n=6), KLH/SWE01 (n=7) and IFN-K/SWE01 (n=7). NT, PBS and KLH-receiving mice were considered as Controls of IFN-K-treated mice.

Except for NT mice, 8-weeks-old-mice received 5 i.m. injections (0.1 mL) of PBS, KLH (10 µg) or IFN-K (10 µg), emulsified in the appropriate adjuvant at a ratio of 1:1, at day 0 (i.e. 54^th^ day of life), 7, 28, 56 and 84.

Blood samples were collected in all mice by retro-orbital bleeding, at day -11 (pre-immune), 38, 66, 94 and 122 (final bleed). At final bleed, surviving mice were euthanized and their organs were harvested.

The stimulated salivary flow rate measurement, phenol red thread test, Von Frey test, cold plate assay, open field test and forced swim test were all performed 2 weeks before, and 8 and 16 weeks after immunization. The sucrose consumption test was performed 1 week before, and 9 and 17 weeks after immunization.

### Anti-IFNα/KLH Titer Assay

Serum anti-IFNα and anti-KLH antibody titers were determined by ELISA, and tested in duplicate, as previously described (19). Briefly, pre-coated ELISA plates with 100 ng of murine IFNα3 (PBL Assay Science, Piscataway, NJ) per well or 100 ng of KLH per well were incubated with serial dilutions of the collected sera. Specific IgG were detected by using anti-mouse IgG-HRP Conjugate (Zymed, 61-6520). Titers were calculated by interpolating the O.D.max/2 on the x axis and the equation used was y=ax+b from the straight line passing by the two points surrounding the O.D.max/2. Titers were expressed as serum dilutions.

### IFNα Neutralizing Capacity Bioassay

The collected sera capacity to neutralize antiviral activity of IFNα was assessed by using the L929 cells with Encephalomyocarditis virus (EMCV) cytopathic effect inhibition assay, as previously described (19). Briefly, sera were tested in duplicate, diluted in assay medium (RPMI supplemented with 4% FBS 2 mM glutamine, 1 mM Sodium Pyruvate, and 1 mM HEPES) and filtered on spinX 0.22 µm tubes. Two-fold serum sample dilution or anti-murine IFNα polyclonal antibody (PBL Assay Science, Piscataway, NJ) as a positive control, and murine IFNα3 (10 U/well) were added to the wells and incubated for 1 h at room temperature. Then, 20.000 L929 cells were added per well and plates were incubated for 18h at 37°C and 5% CO2 in a humidified incubator. After medium removal and PBS washes, the cell culture was infected with EMCV (diluted to 100 Cell Culture Infectious Dose 50). Plates were incubated for 48h until cell survival determination with a 3-(4,5-dimethylthiazol-2-yl)-5-(3-carboxymethoxyphenyl)-2-(4-sulfophenyl)-2H-tetrazolium/phenazine methosulfate (MTS/PMS) solution dye (Promega; Madison, Wisconsin, USA) according to the manufacturer’s instructions. The 50% neutralization capacity (NC50) value (i.e. serum dilution resulting in 50% neutralization of virus-mediated cell mortality) was assessed.

### Anti-IFNα B-Cell Response Assay

Mouse IgG ELISpot^BASIC^ (Mabtech, France) was used for the quantification of anti-IFNα-IgG-producing cells among splenocytes (300 000 cells/well), under different stimulation conditions (no stimulation, Concanavalin A, KLH, IFNα or IFN-K, at a final concentration of 10µg/ml), tested in duplicates. The assays were performed according to the manufacturer’s protocol and an automated reader (BIOSYS, Germany) was used.

### SjS-Related Autoantibodies

Mice sera collected at -11 (1.5 months of age) and 122 days (6 months of age) were used for ELISA detection of autoantibodies and tested in duplicate. Briefly, precoated ELISA plates with murine Ro52 or Ro60 were incubated with 100-fold diluted sera. Specific IgG were detected with HRP-conjugated anti-mouse IgG and binding quantified using 3,3,5,5’-tetramethylbiphenyl-4,4’-diamine (TMB) substrate by absorbance measurement at 490 nm. Threshold for positivity was determined as the mean titer + 5 standard deviations in 6-month-old C57Bl/6 mice (n=5).

### Salivary Flow Rate Measurement

Mice were anesthetized (i.p. injection of a 60mg/ml ketamine and 8mg/ml xylazine solution), and their stimulated (s.c. pilocarpine hydrochloride at 0.5 µg/g of body weight; Santa Cruz Biotechnologies, Dallas, USA) 10-minute-Salivary Flow Rates (SFR) were measured. During anesthesia, saliva was collected continuously for 10 min with a hematocrit capillary tube (Siemens Healthcare Diagnostics, Tarrytown, USA) and transferred to a 1.5 ml tube. After a brief centrifugation, the volume of saliva was determined using a micropipette and standardized against the weight (in grams) of the individual mouse.

### Phenol Red Thread Test

Following saliva collection, phenol red threads (Danyang Nuorishi Optical Co., Jiangsu, China) were used to measure tear production in mice, as previously described (31). Briefly, the bent end of a thread was carefully placed at the intercanthus of one eye and held in place with sterile forceps for 20 seconds. On removal of the thread, the length of the red area was measured (in millimeters), using the scale provided, and recorded.

### Von Frey Test

Tactile sensitivity was evaluated using the von Frey test (32). The principle of this test is standardized. Once the mouse is calm and motionless, a hind paw is touched with the tip of a flexible fiber of given length and diameter (Bioseb, Vitrolles, France). The force of application increases as long as the investigator is pushing the probe and until the fiber bends. This principle makes it possible for the investigator to apply a reproducible force to the skin surface. The scale of force used ranged from 0.008 to 1 g. The force required to induce a withdrawal paw movement was recorded.

### Cold Plate Assay

Cold hypoalgesia was evaluated during 5 min using a cold plate set at 2 ± 0.2°C (Bioseb, Vitrolles, France) as previously described (33). The total number of brisk lifts of either hind paw was counted, and two observers were tasked with counting to ensure accuracy. Normal locomotion was distinct, involving coordinated movements of all four limbs that were excluded. A maximal cut-off time of 5 min was used to prevent tissue damage.

### Open Field Test

To evaluate anxiety-like behavior (34), mice were placed in a field of 40 x 20 cm (surrounded by walls to prevent escape) including a new object in the center, for 10 min. The amount of time the mouse spent in the arena center (15 x 7.5 cm around the new object, i.e. the “exposed field”) and in the arena periphery (the rest of the field, i.e. the “protected field”) were measured using an automated video-tracking device (Smart, Panlab Harvard Apparatus, Barcelona, Spain). Data recorded during the last 5 min were analyzed.

### Forced-Swim Test

To evaluate depressive-like behavior (34), mice were placed in Plexiglas cylinders (20 cm high, 15 cm in diameter) filled with 35°C water to a depth of 15 cm (preventing mice from touching the bottom of the cylinder). During 6 min, the duration of immobility (i.e. the time during which mice made only the smallest movements necessary to keep their heads above water) was scored using an automated video-tracking device and the last 4-minute-data were analyzed.

### Sucrose Consumption Test

Because reduced sensitivity to palatable stimulus is considered to reflect anhedonia in mice (34), their responsiveness to a 4% sucrose solution was evaluated. Three days prior to the beginning of the test, mice were placed in individual cages. Water (1^st^ phase of the test), then 4% sucrose solution (2^nd^ phase) intakes were measured (by reweighting pre-weighted bottles) over two 48h-periods, and average sucrose solution/water intake ratios were analyzed.

### Lymph Nodes Measurement

To evaluate their level of lymphoproliferation, mice’s main lymph nodes (cervical, axillary, brachial and inguinal areas) were measured before sacrifice. A semi-quantitative gradation was performed (0, not palpable; 1, ≤5mm; 2, >5mm).

### Dipstick Test for Proteinuria

Mice were observed for proteinuria at weeks 0, 3, 6, 9, 12 and 16 from first immunization, by using urine dipstick tests (Siemens Healthcare Diagnostics, Tarrytown, USA) which allowed a semi-quantitative gradation, according to the manufacturer’s instructions (0, negative to traces; 1: 0.3 g/l; 2: 1 g/l; 3: ≥3 g/l).

### Murine Sjögren’s Syndrome Disease Activity Index (MuSSDAI)

Modelled on the EULAR Sjögren’s Syndrome Disease Activity Index (35) (ESSDAI), MuSSDAI was designed as a global activity score using all the applicable features in MRL/lpr mice. The weight of each organ-specific domain in MuSSDAI was chosen to be as close as possible to the human index.

Lymphadenopathy (weight = 4) and renal (weight = 5) domains’ scores respectively ranged from 0 to 2 and 0 to 3, using the aforementioned semi-quantitative gradation.

For the other selected domains (glandular, peripheral nervous system [PNS] and central nervous system [CNS]), the results of their respective tests were converted into a semi-quantitative gradation system ranging from 0 (no activity) to 3 (high activity). The limits of each activity class were determined, using the tests’ results in all 52 premorbid MRL/lpr mice, as follows: no activity (=0) when the result was included in the 95% confidence interval (CI95) of the mean; low activity (=1) when the result was between the upper (length of the fibers [Von Frey] and % of immobility [forced-swim test]) or the lower (SFR, phenol red thread test, cold plate assay, % in the center zone [open field test] and sucrose solution/water intake ratio) limit of the CI95 and the same limit ± 15% of the mean; moderate activity (=2) when the result was between the upper/lower limit of the CI95 ± 15% of the mean and the same limit ± 30% of the mean; high activity (=3) when the result was beyond the upper/lower limit of the CI95 ± 30% of the mean.

Glandular domain’s weight (=3) was equally divided between salivary and lacrimal functions assessments. Similarly, PNS (=5) and CNS (=6) domains’ weights were respectively and equally divided between mechanical and thermal nociception evaluations, and between depressive-like behavior, anxiety-like behavior and anhedonia evaluations.

MuSSDAI was calculated for each mouse as the sum of every organ-specific domain’s score multiplied by its own weight and ranged from 0 to 65.

### Histology

Submandibular glands (SMG), lacrimal glands (LG), lungs and kidneys harvested at the time of sacrifice were fixed in 10% neutral buffered formalin and embedded in paraffin wax. Kidneys (4 µm sections) were stained with periodic acid/Schiff light green. SMG, LG and lungs (6 µm sections) were stained with hematoxylin/eosin. For each sample, 2 to 6 sections of both (left and right) organs were blindly examined.

For SMG and LG, the focus score (36), based on enumeration of the number of foci (i.e. infiltrates of at least 50 mononuclear cells/mm²), was determined. For lungs, perivascular (medium to large vessels surrounded by inflammatory cells/total number of medium to large sized vessels) and peribronchiolar (medium to large bronchioles surrounded by inflammatory cells/total number of medium to large bronchioles) infiltrates were scored (37).

### Immunohistochemistry

Tissue sections of formalin-fixed, paraffin-embedded SMG were immersed in a Proteinase K (Euromedex, France) antigen retrieval solution at 5 µg/ml for 10 min at 37°C. Endogenous peroxidase activity was quenched with 3% H_2_O_2_ in TB. Non-specific binding was blocked using 1% Bovine Serum Albumin (BSA) in PBS for 1 hour 40 min at 4°C. Tissues were first incubated with rabbit anti-mouse BAFF (ALX-210-799, Enzo Life Sciences, France) at 10 µg/ml or rabbit isotype control (1:200) antibodies overnight at 4°C; they were then stained with HRP-conjugated goat anti-rabbit secondary antibody (Abcam, Cambridge, UK) for 30 min at room temperature. Staining was developed using 3,3’-diaminobenzidine (DAB) chromogen. Tissue sections were counterstained with hematoxylin. For quantification of local epithelial production of BAFF in SMG, positive zones (brown spots) were blindly counted out of 3 non-overlapping microscope fields (x40) per sample (from IFN-K and KLH groups; n=5 per group).

### Interferon-Inducible Genes Expression Profiling

RNeasy Protect Mini Kit (Qiagen, Maryland, USA) was used for RNA extraction of mandibular lymph nodes, according to the manufacturer’s instructions. Quality was assessed using an Agilent 2100 Bioanalyzer (Agilent Technologies, Santa Clara, USA).

The Mouse Type 1 Interferon Response RT² Profiler PCR Array, RT² First Strand Kit and RT² Real-Time SYBR Green PCR Master Mix (Qiagen, Maryland, USA) were used according to the manufacturer’s instructions and using the data analysis web portal at http://www.qiagen.com/geneglobe. Quantitative real-time PCR array was performed (using 0.5 µg of extracted RNA/mouse) on the ABI 7500 Real-Time PCR system (Applied Biosystems, Foster City, USA). Samples were assigned to Controls (NT, PBS and KLH mice) and test (IFN-K-treated mice) groups. CT values were normalized based on IFNA4 expression (automatically selected from the full panel because of its stable expression). Fold change/regulation was calculated using ΔΔCT method (38), with a cut off of 2.

### Statistics

Means (± standard error of the mean) of MuSSDAI, SFR, phenol red thread, Von Frey, cold plate, sucrose consumption, forced-swim and open field tests, as well as histological scoring were analyzed between control (NT, PBS and KLH groups) and treated (IFN-K) mice by the Mann-Whitney U test. These parameters and cells’ response assays results were also analyzed by the Kruskal-Wallis test, mainly to assess the lack of differences between the 3 control groups, followed by Dunn tests to compare individually the treated groups with their respective control groups. Survival and proteinuria data were analyzed using Kaplan–Meier graphs and log-rank tests. For IFN-inducible genes expression, the p values were calculated based on a Student’s t-test of the replicate 2^(-ΔCT)^ values for each gene in the Control and IFN-K-treated groups.

The data analyses were performed using GraphPad PRISM 5.0 (Intuitive Software for Science, USA).

## Results

### Immunogenicity and Neutralization

Immunization with IFN-K adjuvanted with SWE01 or ISA51 (**Figures 1B, C**) efficiently elicited high titers of anti-IFNα Abs in all treated mice by day+38 post immunization (respectively 1/27172 ± 1/2856 and 1/16768 ± 1/3861, p=0.0973) with persistent titers at day+66 (1/25631 ± 1/2674 and 1/14137 ± 1/3726, p=0.0728) day+94 (1/25386 ± 1/3718 and 1/8068 ± 1/2197, p=0.0047) and day+122 (1/16313 ± 1/2304 and 1/9654 ± 1/2963, p=0.1014). KLH/ISA51-treated mice belatedly developed low titers of anti-IFNα Abs starting at day+94 (1/1682 ± 1/492) and still present at day+122 (1/1643 ± 1/308), without detectible neutralizing properties.

Neutralizing anti-IFNα Abs were only detected in IFN−K-treated mice, with higher neutralizing capacities in the IFN-K/SWE01 group than in the IFN-K/ISA51 group (**Figure 1D**), at all time points: day+38 (1/287 ± 1/61 versus 1/56 ± 1/36, p=0.0095), day+66 (1/2226 ± 1/799 versus 1/147 ± 1/77, p=0.0045), day+94 (1/2168 ± 1/663 versus 1/151 ± 1/59, p=0.0079) and day+122 (1/2363 ± 1/548 versus 1/95 ± 1/44, p=0.0032).

The quantification of anti-IFNα-IgG-secreting splenocytes (see **Supplementary Figure S1** for the detailed analysis) was superior in IFN-K/SWE01-treated mice (**Figure 1E**) than in their controls (183 spots ± 11 versus 31 spots ± 4, p=0.0079), regardless of the restimulation conditions. The difference was not significant between IFN-K/ISA51-treated mice (**Figure 1F**) and their controls (32 spots ± 3 versus 26 spots ± 1, p=0.1508).

Only mice that received KLH (i.e. KLH and IFN-K groups) developed anti-KLH Abs (see **Supplementary Figure S2**)

### Type 1 IFN Signature

**Figure 2:**
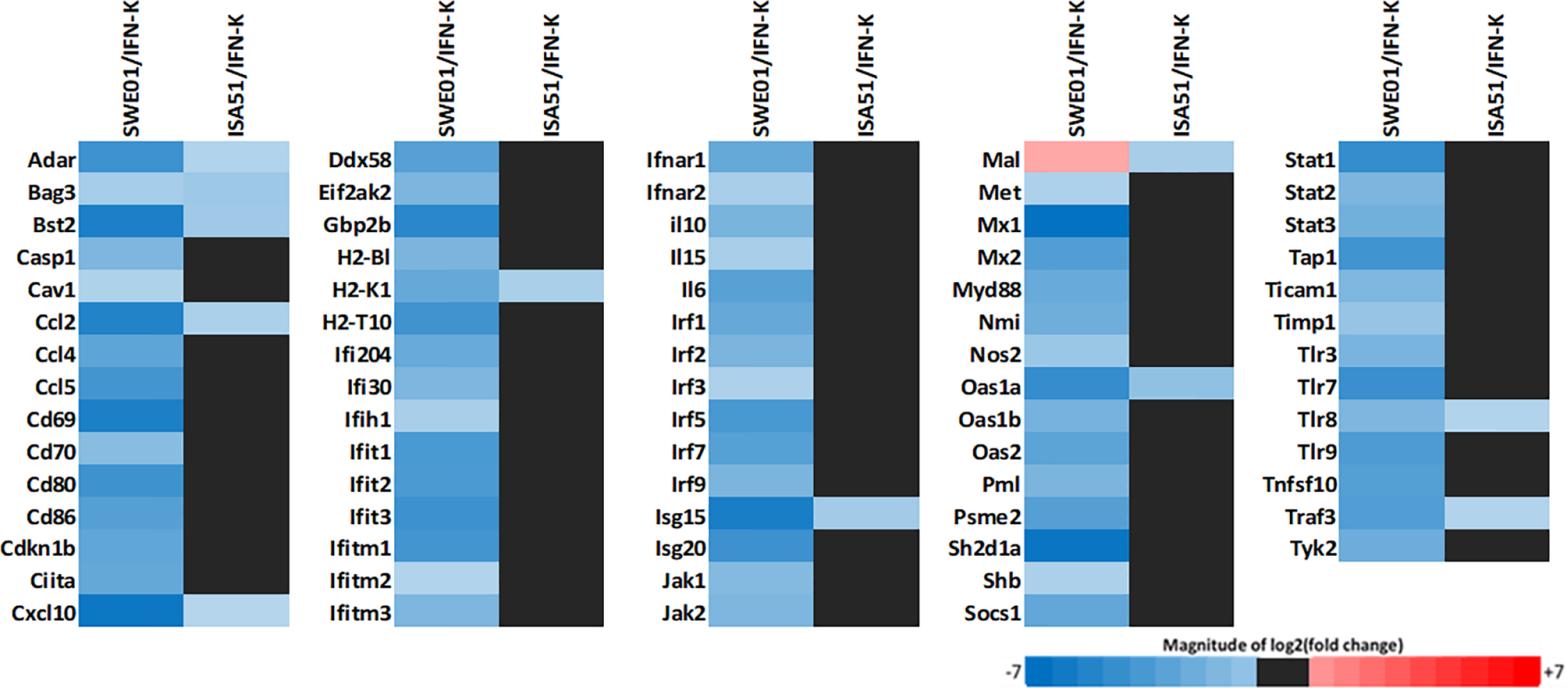
Type 1 IFNs signature. Mandibular lymph nodes were isolated in all living mice (n=14 in SWE01-adjuvanted control mice, n=6 in SWE01/IFN-K mice, n=18 in ISA51-adjuvanted control mice and n=7 in ISA51/IFN-K mice) at sacrifice (D+122) to determine the expression profile of type 1 IFNs pathways genes (type 1 IFN signature). Fold changes were determined using the ΔΔCT method, with a cut off of 2, and compared between IFN-K-treated mice (SWE01/IFN-K and ISA51/IFN-K) and their controls (not treated, PBS and KLH groups).

Type 1 IFN-inducible genes expression (**Figure 2** and see **Supplementary Figure S3** for a more detailed analysis) was reduced in IFN-K-treated mice’s mandibular lymph nodes when compared to their controls, with a superior effect with SWE01. Seventy-three transcripts were downregulated (with log2[fold change] ranging from -6.9 to -2.11) in the IFN-K/SWE01 group, and 10 (with log2[fold change] ranging from -3.02 to -2.01) in the IFN-K/ISA51 group. Among the more downregulated transcripts in the IFN-K/SWE01 group were classical IFN-inducible genes: *Mx1* (-6.9), *Cxcl-10* (-6.62), *Isg15* (-6.27), *Ccl2* (-6.02), *Stat1* (-5.57), *Oas1a* (-5.54), or *Tlr7* (-5.4). Among these transcripts, *Cxcl-10*, *Isg15*, *Ccl2*, and *Oas1a* were downregulated in both IFN-K-treated groups.

### Global Disease Activity

**Figure 3:**
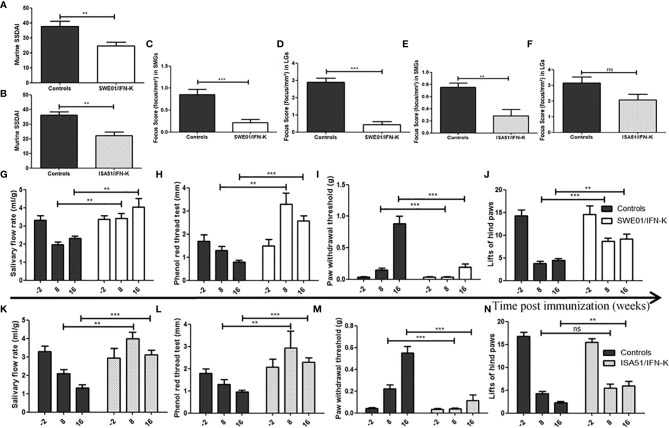
Improving Sjögren’s syndrome in MRL/lpr mice. Sjögren’s syndrome-like features were monitored and compared between IFN-K-treated mice and their controls (not treated, PBS and KLH groups). **(A, B)** Modeled on the EULAR Sjögren’s Syndrome Disease Activity Index (ESSDAI), we designed a Murine Sjögren’s Syndrome Disease Activity Index (MuSSDAI), using all the applicable features in MRL/lpr mice. MuSSDAI was assessed at the end of follow-up (n=20 in SWE01 cohort and n=25 in ISA51 cohort), as was determined **(C–F)** the focus score, i.e. the degree of lymphoid infiltration (one focus = an aggregate of ≥ 50 mononuclear cells), in **(C, E)** submandibular and **(D, F)** lacrimal glands. **(G, K)** Salivary flow rates, **(H, L)** tear production, **(I, M)** mechanical and **(J, N)** thermal sensitivities were assessed 2 weeks before immunization (n=26 in both cohorts), 8 weeks (n=26 in both cohorts) and 16 weeks (n=21 in SWE01 cohort and n=25 in ISA51 cohort) post-immunization. Error bars indicate mean ± S.E.M. Ns **p < 0.01 and ***p < 0.001. SMG, submandibular glands; LG, lacrimal glands.

The Murine Sjögren’s Syndrome Disease Activity Index (MuSSDAI), a systemic disease activity score we modelled on the EULAR Sjögren’s Syndrome Disease Activity Index (ESSDAI) (**Supplementary Table S1**), was statistically improved in IFN-K/SWE01 (24.8 ± 2.3 versus 37.8 ± 3.5, p=0.0059) and IFN-K/ISA51-treated (22.1 ± 2.6 versus 36.2 ± 2.2, p=0.0012) mice when compared to their respective controls (**Figures 3A, B**).

### Glandular Features

IFN-K improved saliva and tear productions in MRL/lpr mice, when compared to control groups. In SWE01-adjuvanted groups, the SFR (**Figure 3G**) were 3.4 ± 0.8 ml/g versus 2.0 ± 0.7 ml/g (p=0.0026) and 4.0 ± 1.2 ml/g versus 2.3 ± 0.5 ml/g (p=0.0044), while the phenol red thread tests (**Figure 3H**) were 3.3 ± 0.5 mm versus 1.3 ± 0.2 mm (p=0.0017) and 2.6 ± 0.2 mm versus 0.8 ± 0.1 mm (p=0.0002), at 8 and 16 weeks post-immunization, respectively. In ISA51-adjuvanted groups, the SFR (**Figure 3K**) were 4.0 ± 0.4 ml/g versus 2.1 ± 0.2 ml/g (p=0.0012) and 3.1 ± 0.3 ml/g versus 1.3 ± 0.2 ml/g (p=0.0004), and the phenol red thread tests (**Figure 3L**) were 2.9 ± 0.8 mm versus 1.3 ± 0.2 mm (p=0.0027) and 2.3 ± 0.2 mm versus 0.9 ± 0.1 mm (p=0.0001) at the same time points.

**Figure 4:**
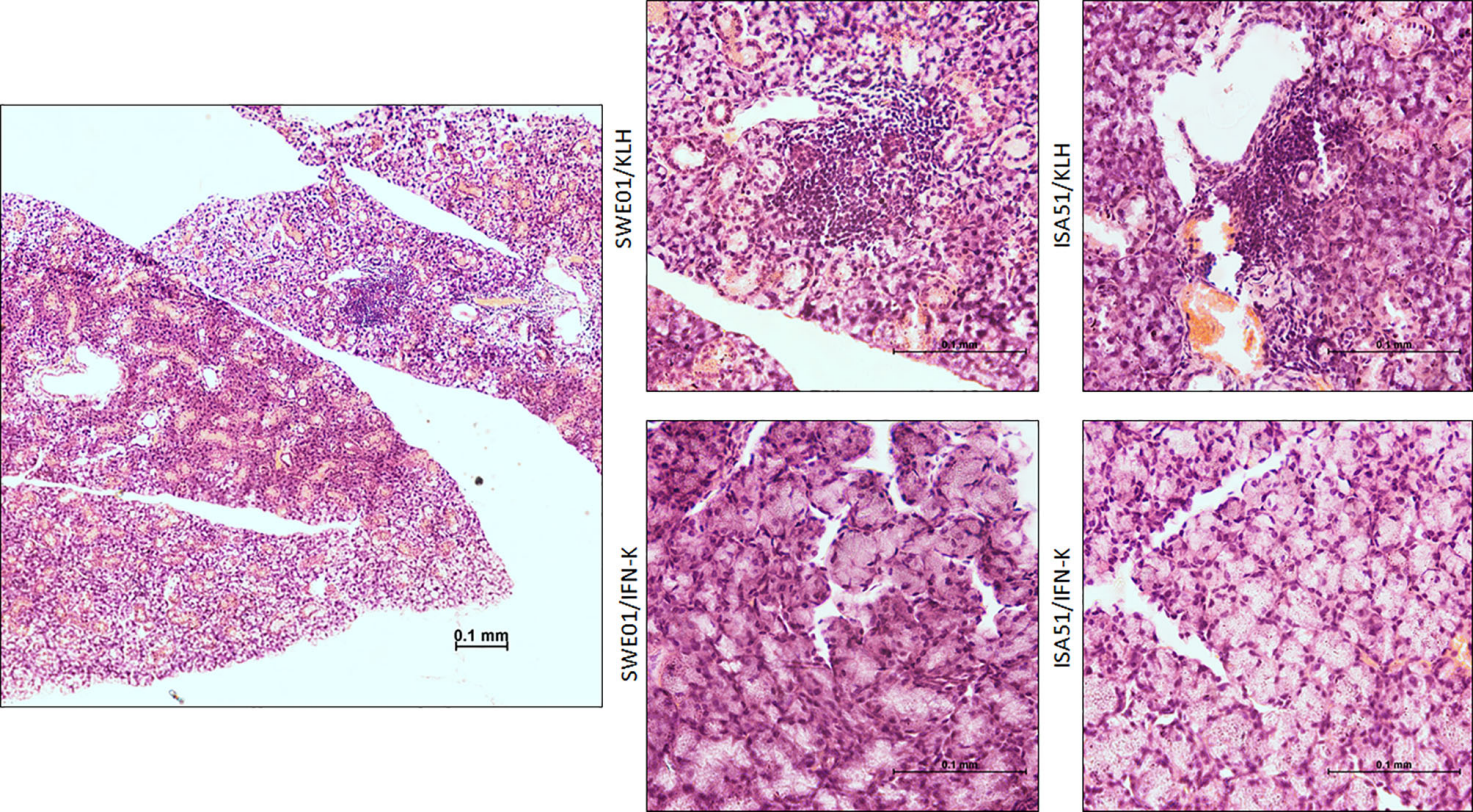
Salivary histopathological features. Representative low (left; x10) and high (right; x40) magnification images of hematoxylin and eosin-stained sections of the submandibular glands harvested at the end of follow-up in SWE01/KLH (n=5), SWE01/IFN-K (n=6), ISA51/KLH (n=7) and ISA51/IFN-K (n=7) living mice, are shown.

**Figure 5:**
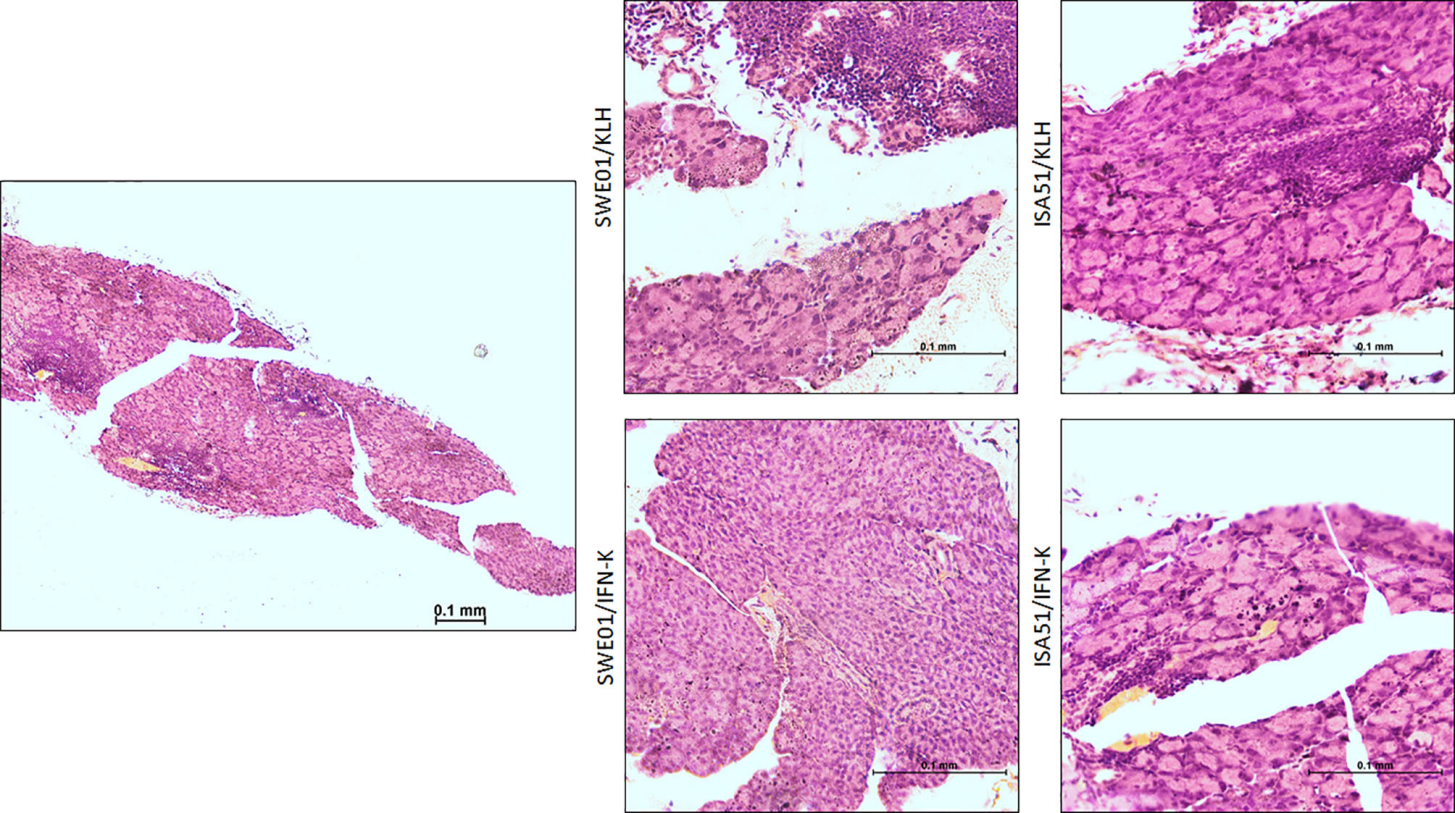
Lacrimal histopathological features. Representative low (left; x10) and high (right; x40) magnification images of hematoxylin and eosin-stained sections of the lacrimal glands harvested at the end of follow-up in SWE01/KLH (n=5), SWE01/IFN-K (n=6), ISA51/KLH (n=7) and ISA51/IFN-K (n=7) living mice, are shown.

**Figure 6:**
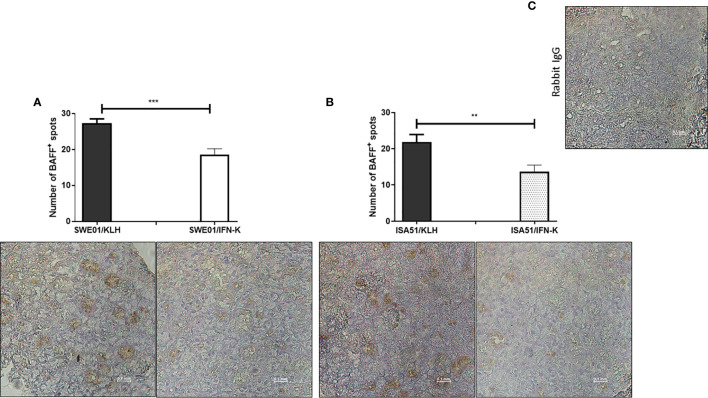
In-situ reduction of B cell activating factor (BAFF) production. Local expression of BAFF was analyzed by immunochemistry in formalin-fixed, paraffin-embedded submandibular glands (SMG) harvested at the end of follow-up in SWE01/KLH, SWE01/IFN-K, ISA51/KLH and ISA51/IFN-K mice (n=5 per group). BAFF-positive zones (brown spots) in SMG were counted out of 3 non-overlapping microscope fields (x40) per sample and compared between controls and treated mice in **(A)** SWE01 cohort and **(B)** ISA51 cohort. Representative images of BAFF-immunostained sections and **(C)** negative control (rabbit IgG) are shown (original magnification x40). Error bars indicate mean ± S.E.M. **p < 0.01 and ***p < 0.001.

At the histological level, the focus scores were improved in SMG (0.22 focus/mm² ± 0.07 versus 0.85 focus/mm² ± 0.12, p=0.0010) and LG (0.44 focus/mm² ± 0.17 versus 2.90 focus/mm² ± 0.23, p=0.0006) of IFN-K/SWE01-treated mice (**Figures 3C, D**), when compared to SWE01-adjuvanted controls. The same beneficial effect was observed in IFN-K/ISA51-treated mice’s SMG (0.28 focus/mm² ± 0.10 versus 0.75 focus/mm² ± 0.07, p=0.0068), but in their LG (2.08 focus/mm² ± 0.35 versus 3.15 focus/mm² ± 0.38, p=0.0602) the downward trends did not reach statistical significance (**Figures 3E, F**). Representative microphotographs are shown in **Figures 4** for SMG, and **Figure 5** for LG. Accordingly, immunochemistry experiments showed a significant reduction in immunostaining for BAFF in SMG from IFN-K-treated mice when compared to SMG from KLH-treated mice (**Figure 6**) in SWE01 (18.5 spots ± 1.6 versus 27.3 spots ± 1.3, p=0.0003) as was as in ISA51 (13.7 spots ± 1.8 versus 21.9 spots ± 2.1, p=0.0099) cohorts (**Figure 4**).

### Extra-Glandular Features

Mechanically-induced nociception was not altered in IFN-K-treated mice when compared to mice from the control groups whose hypoesthesia significantly worsened with aging. In SWE01-adjuvanted groups (**Figure 3I**), the threshold of withdrawal of the touched paw was lower in IFN-K-treated mice than in their controls, at 8 weeks (0.03 ± 0.01 g versus 0.15 ± 0.03 g, p=0.0007) and 16 weeks (0.19 ± 0.06 g versus 0.89 ± 0.12 g, p=0.0005) post-immunization. In ISA51-adjuvanted groups (**Figure 3M**), the threshold of withdrawal of the touched paw was also lower in IFN-K-treated mice than in their controls, at 8 weeks (0.04 ± 0.01 g versus 0.22 ± 0.04 g, p=0.0004) and 16 weeks (0.11 ± 0.05 g versus 0.55 ± 0.06 g, p=0.0006) post-immunization.

As for thermally-induced nociception (**Figures 3J, N**), earlier effects were observed in IFN-K/SWE01-treated mice (with a higher mean number of brisk lifts of either hind paw), starting at 8 weeks (8.71 ± 0.68 paw lifts versus 3.74 ± 0.52 paw lifts, p=0.0005) post-immunization and maintained at 16 weeks (9.14 ± 1.14 paw lifts versus 4.43 ± 0.48 paw lifts, p=0.0016)., when beneficial effects were also significant in IFN-K/ISA51-treated mice (6.00 ± 0.98 paw lifts versus 2.32 ± 0.30 paw lifts, p=0.0021). No difference was observed between IFN-K/ISA51-treated mice and their controls at 8 weeks post-immunization (5.43 ± 0.95 paw lifts versus 4.32 ± 0.47 paw lifts, p=0.41).

**Figure 7:**
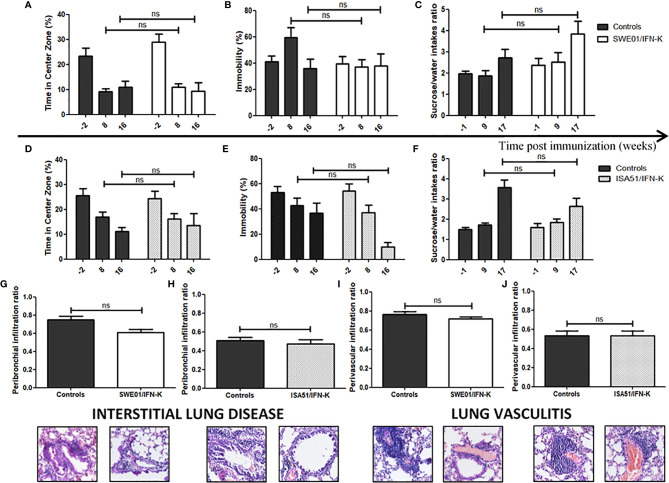
Unaffected features of systemic Sjögren’s syndrome in MRL/lpr mice. **(A, D)** Anxiety-like (leading to a reduction of the time spent in the center zone in the open field) and **(B, E)** depression-like (leading to a higher degree of immobility in the forced swim test) behaviors were assessed 2 weeks before immunization (n=26 in both cohorts), 8 weeks (n=26 in both cohorts) and 16 weeks (n=21 in SWE01 cohort and n=25 in ISA51 cohort) post-immunization with **(A, B)** SWE01 or **(D, E)** ISA51 as adjuvant. **(C, F)** Anhedonia, leading to a reduction in sucrose/water intakes ratios, was assessed before immunization (-1 week; n=26 in both cohorts), 9 weeks (n=25 in SWE01 cohort and n=26 in ISA51 cohort) and 17 weeks (n=20 in SWE01 cohort and n=25 in ISA51 cohort) post-immunization. Lungs were harvested for all living mice (n=20 in SWE01 cohort and n=25 in ISA51 cohort) at the time of sacrifice (D+122), to evaluate the extent of their pulmonary disease. **(G, H)** The peribronchiolar infiltration ratio (i.e. the number of medium to large bronchioles surrounded by inflammatory cells over the total number of medium to large bronchioles on each slide) and the **(I, J)** perivascular infiltration ratio (i.e. the number of medium to large vessels surrounded by inflammatory cells over the total number of medium to large sized vessels on each slide) were calculated to quantify the degree of interstitial lung disease and lung vasculitis, respectively. Representative images of hematoxylin and eosin stained sections are shown (original magnification x40). No IFN-K-related effect was observed when comparing the treated mice and their controls (NT, PBS and KLH mice). Error bars indicate mean ± S.E.M. Ns p > 0.05.

Neuropsychiatric tests and lung infiltrated were not affected by IFN-K (**Figure 7**).

Proteinuria-free survival (**Supplementary Figure S4**) was not statistically different between groups.

Anti-Ro60 and anti-Ro52 autoantibodies (**Supplementary Figure S5**) were respectively positive in 44.4% and 68.9% of the 1.5-month-old mice, and 100% of the 6-month-old mice, but they were not statistically different between groups.

## Discussion

IFN-K induced high levels of anti-IFNα Abs, regardless of its adjuvantation protocol, with higher trends in IFN-K/SWE01-treated mice. However, the difference was only significant at one time point. In both IFN-K-vaccinated groups, both glandular and PNS manifestations of the disease were significantly improved. These improvements were concordant at the histological level, as shown by the reduced inflammatory cell infiltrates in the glands of IFN-K-treated mice. In IFN-K/ISA51-treated mice, a similar beneficial effect was observed, but did not reach statistical significance in lacrimal glands, which is although not incompatible with a normalized tear production (39).

Regarding the anti-IFNα neutralizing capacity, there was a clear superiority in IFN-K/SWE01-treated mice, with concordant results in the large downregulation of type 1 IFN signature and the quantification of anti-IFNα-IgG-secreting splenocytes. As a MF59-like adjuvant, SWE01’s superiority may be due to its known positive effect on T follicular helper cells’ (TFH) response and thus the magnitude of the germinal center B cell response (40). MF59 has previously been shown to be able to induce a greater adjuvant efficacy (with different vaccine antigens: influenza, tetanus toxoid, hepatitis B, Group B and C Meningococcal bacteria) than any other adjuvant (41), including ISA51 (42).

The fact that KLH/ISA51-treated mice belatedly developed low titers of non-neutralizing-anti-IFNα Abs starting at day+94, and that all control mice had low counts of anti-IFNα-IgG-secreting splenocytes, indicate that, IFN-K induces an anti-IFNα Abs production by increasing or promoting the maturation of pre-existing autoreactive B cells as previously described in SLE patients (20, 43).

As for the other extra-glandular manifestations, the neuropsychiatric manifestations of the disease were not affected by IFN-K, but the behavioral tests evaluating these features are known to be complex with many confounding factors (44). Furthermore, proteinuria-free survival was also not different between groups. However, our trial was not specifically designed to evaluate this aspect of the disease (because glomerulonephritis in the MRL/lpr model is more a lupus-like feature than a SjS-like one) and the occurrence of these events was lower than expected (45). Finally, about the fact that pulmonary disease was unaffected by IFN-K, we think that a non-IFN-dependent pathologic process might be mediating lung damage, as it has previously been improved in MRL/lpr mice by inhibiting Tumor Necrosis Factor-α (37). This element will have to be kept in mind during IFN-K development.

The main limitation that one could see in this study is the use of the MRL/lpr mouse model, because it is usually referred to as a secondary mouse model of SjS. That is because it presents with glomerulonephritis (which was absent in our mice) and CNS manifestations, which are rare but not absent in SjS (4). However, many authors (46–48) now criticize the primary/secondary SjS dichotomy which misrepresents the potential manifestations and risks of the syndrome. As Professor Moutsopoulos (among others), who coined the concept of secondary SjS more than 35 years ago, we think that the term “secondary”, in human and murine SjS, should be replaced by a more descriptive term (*i.e.* “SjS associated with”) or by the term “polyautoimmunity” (47, 48). Moreover, the MRL/lpr mouse model is included in all review papers about murine models of SjS, including the ones focusing on primary SjS and it has all key features of SjS: female sex predilection, decreased salivary flow rate (49) and tear production, lymphocytic infiltrates in the salivary and lacrimal glands, and anti-Ro60, anti-Ro52 and anti-La autoantibodies. On this latter point, we detected a much higher rate of anti-Ro60 and/or anti-Ro52-positive mice than what had been historically described in the MRL/lpr mouse model (6% and 30% respectively) (34, 35), using the same stringent threshold. The two main elements to explain these differences are probably the type of antigens which were used [full-length murine proteins versus partial human proteins for the publication dating from 1994 (50)] and the age of the mice which were tested (6-month-old mice in our study versus 4-5-month-old mice previously). In addition to the rare aforementioned CNS and renal manifestations these mice also develop classical systemic SjS manifestations: peripheral neuropathy (rare in SLE), arthritis, pneumonitis, cryoglobulinemia and signs of lymphoproliferation (lymphadenopathies, splenomegaly) (34). As we noted in a recent correspondence (51), no perfect model for SjS exists, but we think that the MRL/lpr mouse model should be considered as a model of choice for preclinical trials evaluating new immunological treatments in SjS, because of its unique systemic manifestations. Another limitation could be the use of mandibular lymph nodes to evaluate type 1 IFN signature, however, even if we had to use most of SMG tissues to perform the more rigorous and accurate evaluation of the focus score, we were able to demonstrate that local epithelial production of BAFF – which is a pivotal type 1 IFN-inducible cytokine – was reduced in SMG from IFN-K-treated mice compared to their controls’. In line with our findings, other teams have previously reported that type 1 IFNs and plasmacytoid dendritic cells were involved in the pathogenesis of the MRL/lpr mouse model (52). It is also important to note that the draining lymph nodes were previously described as important elements in SjS (53, 54).

Anti-Ro60 or anti-Ro52 titers did not differ between groups, which is consistent with the absence of fluctuation in anti-Ro titers with disease activity in human SjS (55).

As reported in previous preclinical studies (19) no signs of autoreactive anti-IFNα T cell response were detected using IFN-γ ELISpot^BASIC^ (data not shown).

We herein describe for the first time, a proof-of-concept for the efficacy of IFN-K in a mouse model of systemic SjS. Its beneficial effects on sicca syndrome and peripheral neuropathy pave the way for its clinical development in SjS, which is still facing an unmet therapeutic need. Due to its substantial advantages when compared to monoclonal Abs (absence of anti-drug Abs formation, neutralization of all IFNα subtypes (56), better predictable patient compliance because of a low number of administrations (21), lower production and health care costs…) and its good safety profile (20, 21), IFN-K is the good candidate for Sjögren’s syndrome’s treatment, but of course, further studies are required to confirm this proof-of-concept.

## Data Availability Statement

The original contributions presented in the study are included in the article/**Supplementary Material**. Further inquiries can be directed to the corresponding author.

## Ethics Statement

The animal study was reviewed and approved by PLEXAN.

## Author Contributions

MK, GG-V, FB, and SP designed the study. MK, FC, PH, CN, OC, SC, NR, BC, MT, NL and FF conducted experiments. MK acquired data. MK, FC, OC, SC, BC, MT, FF, GG-V, FB, and SP analyzed data. NL, FF, GG-V, FB, and SP provided reagents. MK wrote the manuscript. FC, OC, PC, GG-V, FB, and SP reviewed the manuscript. All authors contributed to the article and approved the submitted version.

## Funding

This work was supported by the Association Française du Gougerot Sjögren (AFGS) and NEOVACS S.A. The funder was not involved in the study design, collection, analysis, interpretation of data, the writing of this article or the decision to submit it for publication.

## Conflict of Interest

Authors FC, PH and GG-V were employed by company NEOVACS S.A.

The remaining authors declare that the research was conducted in the absence of any commercial or financial relationships that could be construed as a potential conflict of interest.

## Publisher’s Note

All claims expressed in this article are solely those of the authors and do not necessarily represent those of their affiliated organizations, or those of the publisher, the editors and the reviewers. Any product that may be evaluated in this article, or claim that may be made by its manufacturer, is not guaranteed or endorsed by the publisher.
