# Supplementary material for: Therapeutic Potential of Anti-Interferon α Vaccination on SjS-Related Features in the MRL/lpr Autoimmune Mouse Model
Source: Front Immunol. 2021 Nov 17;12:666134. doi: 10.3389/fimmu.2021.666134 (PMC8635808; doi:10.3389/fimmu.2021.666134)

**SUPPLEMENTARY TABLES AND FIGURES**

**Supplementary Figure S1. Detailed evaluation of anti-IFNα secreting splenocytes.** Mononuclear splenocytes were isolated in all living mice (n=14 in SWE01-adjuvanted control mice, n=6 in SWE01/IFN-K mice, n=18 in ISA51-adjuvanted control mice and n=7 in ISA51/IFN-K mice) at sacrifice (D+122), and used for the quantification of anti-IFNα-IgG-producing cells. Proliferation of the harvested cells was stimulated under different conditions (no stimulation, ConA, KLH, IFNα or IFN-K, at a final concentration of 10 µg/ml), without noticeable impact. Error bars indicate mean ± S.E.M. Ns p > 0.05, *p < 0.05, **p < 0.01 and ***p < 0.001.


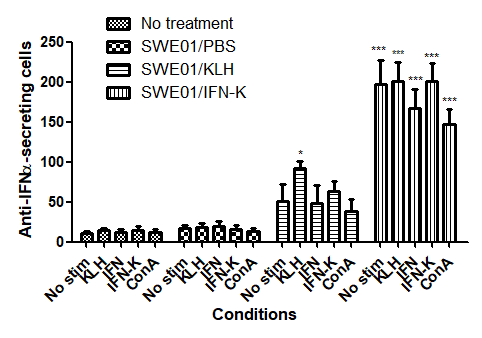

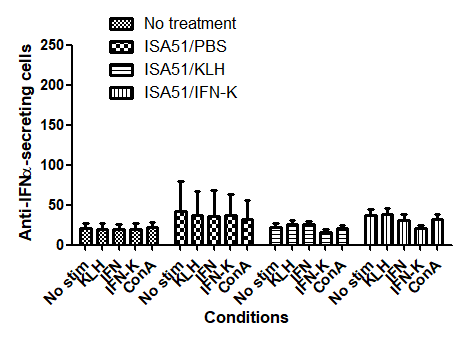


**a**

**b**

**Supplementary Figure S2. Anti-KLH antibody response in MRL/lpr mice.** Serum anti-KLH Abs (ELISA) were monitored at different time points pre (D-11) and post-immunization (D+38, D+66, D+94, D+122) in all groups: no treatment (n=6+6), SWE01/PBS (n=6), ISA51/PBS (n=6), SWE01/KLH (n=7), ISA51/KLH (n=7), SWE01/IFN-K (n=7) and ISA51/IFN-K (n=7).


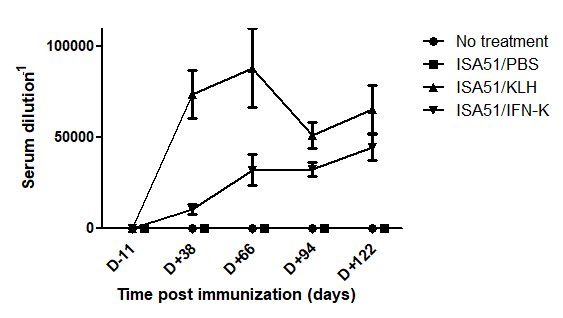

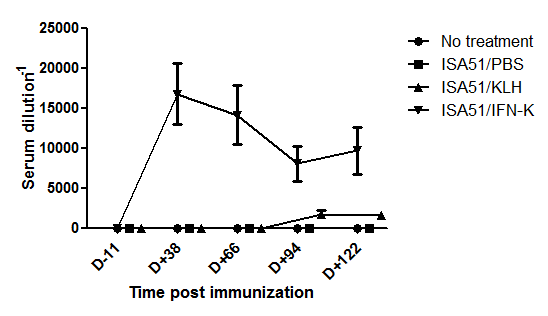


**b**


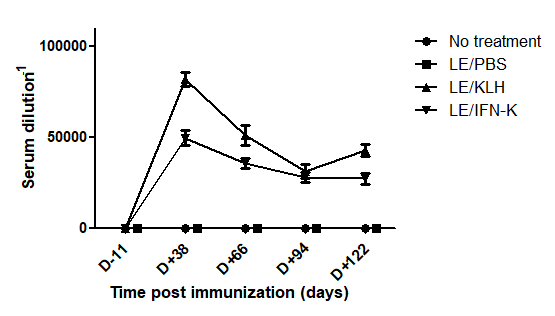


**a**


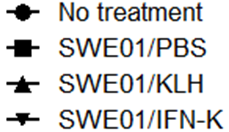


**Supplementary Figure S3. Supplementary data about type 1 IFNs signature.** Mandibular lymph nodes were isolated in all living mice (n=14 in SWE01-adjuvanted control mice, n=6 in SWE01/IFN-K mice, n=18 in ISA51-adjuvanted control mice and n=7 in ISA51/IFN-K mice) at sacrifice (D+122) to determine the type 1 IFN signature. (a) Fold changes were determined using the ΔΔCT method, with a cut off of 2, and compared between IFN-K-treated mice (SWE01/IFN-K and ISA51/IFN-K) and their controls (not treated, PBS and KLH groups). (b) The scatter plots compare the normalized expression of every gene on the array between the two selected groups by plotting them against one another to quickly visualize large gene expression changes. The central line indicates unchanged gene expression. The dotted lines indicate the selected fold regulation threshold. Data points beyond the dotted lines in the upper left and lower right sections meet the selected fold regulation threshold.

**b**

**a**


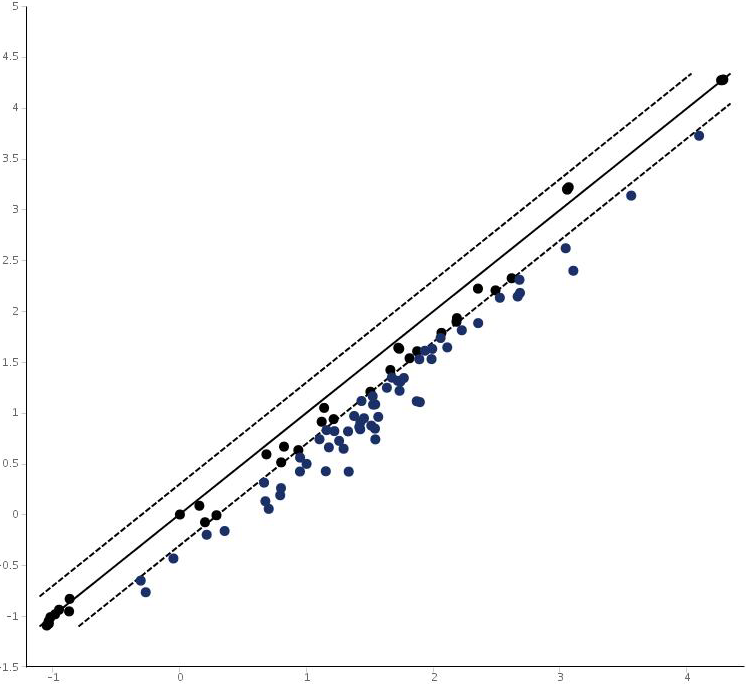

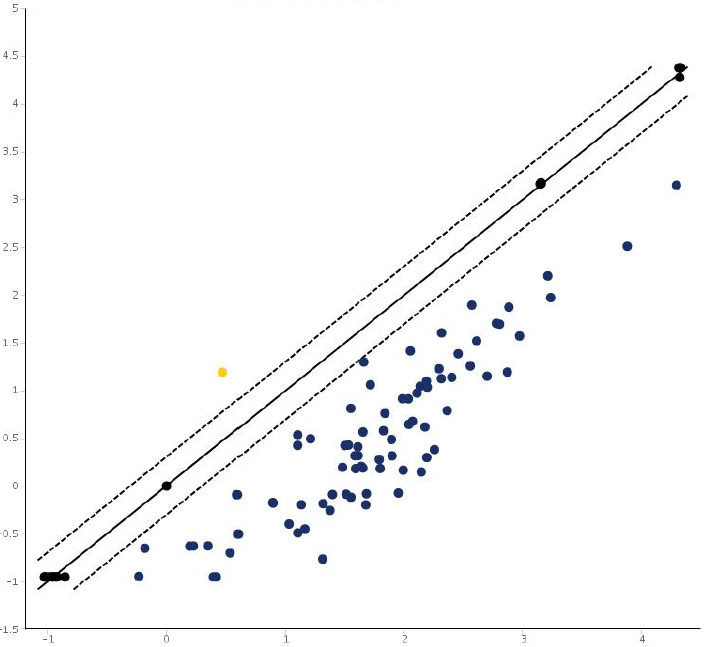


**ISA51/IFN-K** vs **ISA51-adjuvanted controls**

**Log10 (Normalized Expression Control Groups)**

**Log10 (Normalized Expression IFN-K-treated groups)**

**SWE01/IFN-K** vs **SWE01-adjuvanted controls**


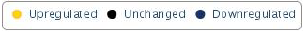


**b**

|  | Weight in | |
| --- | --- | --- |
| Domains | ESSDAI | MuSSDAI |
| Constitutional | 3 | NA |
| Lymphadenopathy | 4 | 4 |
| Glandular | 2 | 3 |
| Articular | 2 | NA |
| Cutaneous | 3 | NA |
| Pulmonary | 5 | NA |
| Renal | 5 | NA |
| Muscular | 6 | NA |
| PNS | 5 | 5 |
| CNS | 5 | 6 |
| Hematological | 2 | NA |
| Biological | 1 | NA |

**Supplementary Table S1. A murine global SS activity score.** Modeled on the EULAR Sjögren’s Syndrome Disease Activity Index (ESSDAI), we designed a Murine Sjögren’s Syndrome Disease Activity Index (MuSSDAI), using all the applicable features in MRL/lpr mice. The weight of each organ-specific domain in MuSSDAI was chosen to be as close as possible to the human index.

**Supplementary Figure S4. Proteinuria-free survival in MRL/lpr mice after IFN-K immunization.** All MRL/lpr mice were monitored for proteinuria (0, 3, 6, 9, 12 and 16 weeks post-immunization) and death was recorded daily. Due to the high impact of severe nephropathy on survival, Kaplan-Meier graphs for (a) SWE01 and (b) ISA51-adjuvanted groups include survival data and severe proteinuria (>3g/l) occurrence under the term “Proteinuria-free survival”. Trends favoring IFN-K emerged, but no statistically significant difference was observed when compared to their controls (NT, PBS and KLH mice).

**d**

**e**


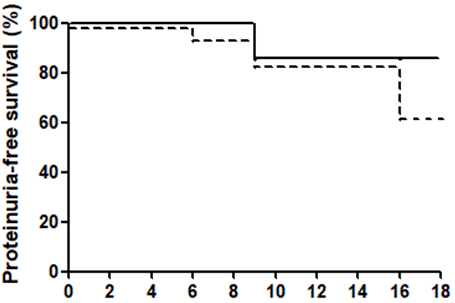

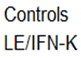

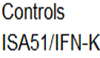

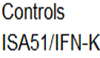

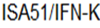


**b**


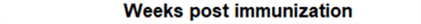

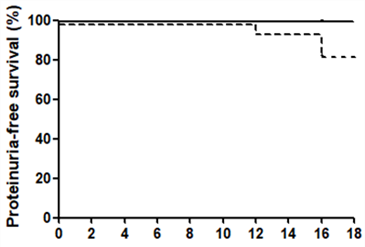


**a**


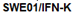

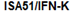


**Supplementary Figure S5. Evaluation of Anti-Ro60 and anti-Ro52 autoantibodies in young and aged MRL/lpr mice.** Hundred-fold diluted sera from (a, c) SWE01 (n=20) and (b, d) ISA51 (n=25) cohorts, collected at 1.5 months of age and 6 months of age, were used to detect (a, b) anti-Ro60 and (c, d) anti-Ro52 autoantibodies. Threshold for positivity was determined as the mean optical density (OD) + 5 standard deviations in 6-month-old C57Bl/6 mice (n=5).

**b**

**a**

**c**

**d**


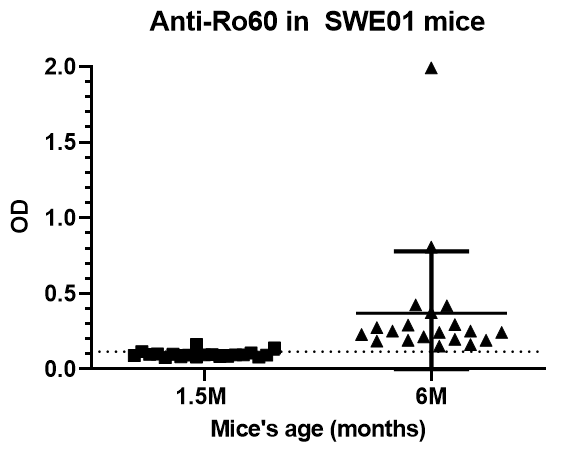

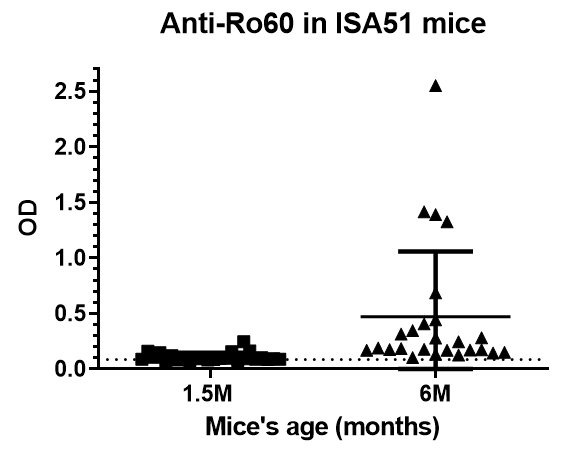

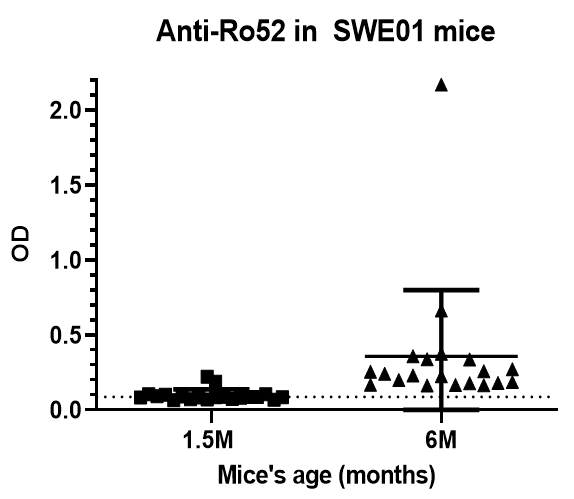

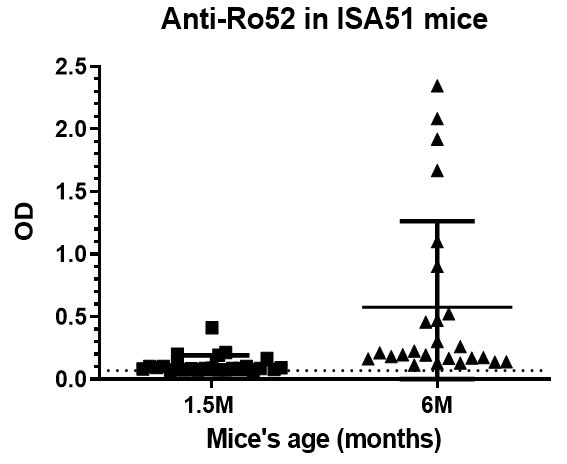

Supplement: Supplementary file 1 [file DataSheet_1.docx]
